# 4-(2,5-Dihexyl­oxyphen­yl)benzoic acid

**DOI:** 10.1107/S1600536808032170

**Published:** 2008-10-11

**Authors:** Hong Li, Lu Zhang, Yan-Qi Liu, Duo-Bin Mao, Wen-Ye Zhang

**Affiliations:** a School of Food and Biological Engineering, Zhengzhou University of Light Industry, Zhengzhou 450002, People’s Republic of China

## Abstract

In the title compound, C_25_H_34_O_4_, one *n*-hexyl chain of the hex­yloxy group adopts a fully extended all-*trans* conformation, and the other *n*-hexyl chain displays disorder with site occupancies of 0.470 (3) and 0.530 (3). The dihedral angle between the benzene rings is 44.5 (3)°. In the crystal structure, inter­molecular O—H⋯O hydrogen bonds form dimers *via* crystallographic inversion centres.

## Related literature

For a review of applications of Suzuki–Miyura cross-coupling reactions in organic syntheses, see: Kotha *et al.* (2002 ▶). For the structure of 1,4-dibromo-2,5-bis­(hex­yloxy)benzene, see: Li *et al.* (2008 ▶). For the syntheses of related compounds, see: Maruyama & Kawanishi (2002 ▶); Zhang *et al.* (2006 ▶).
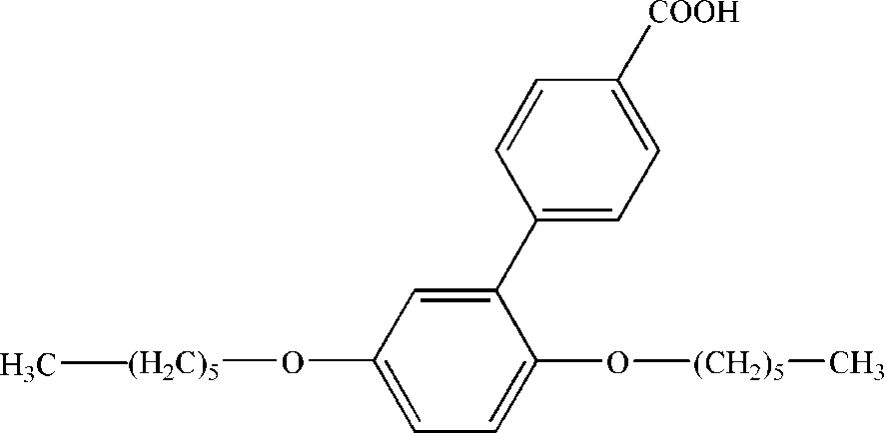


## Experimental

### 

#### Crystal data

C_25_H_34_O_4_
*M* *_r_* = 398.52
Monoclinic, 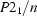

*a* = 7.2936 (12) Å
*b* = 14.689 (2) Å
*c* = 22.137 (4) Å
β = 95.283 (3)°
*V* = 2361.7 (7) Å^3^
*Z* = 4
Mo *K*α radiation
μ = 0.07 mm^−1^
*T* = 295 (2) K
0.35 × 0.15 × 0.06 mm

#### Data collection

Bruker SMART CCD diffractometer
Absorption correction: multi-scan (*SADABS*; Sheldrick, 1996 ▶) *T*_min_ = 0.975, *T*_max_ = 0.996
15777 measured reflections
4359 independent reflections
1432 reflections with *I* > 2σ(*I*)
*R*_int_ = 0.087

#### Refinement

*R*[*F* ^2^ > 2σ(*F* ^2^)] = 0.061
*wR*(*F* ^2^) = 0.194
*S* = 0.97
4359 reflections
257 parameters
H-atom parameters constrained
Δρ_max_ = 0.16 e Å^−3^
Δρ_min_ = −0.14 e Å^−3^

### 

Data collection: *SMART* (Bruker, 2004 ▶); cell refinement: *SAINT* (Bruker, 2004 ▶); data reduction: *SAINT*; program(s) used to solve structure: *SHELXS97* (Sheldrick, 2008 ▶); program(s) used to refine structure: *SHELXL97* (Sheldrick, 2008 ▶); molecular graphics: *SHELXTL* (Sheldrick, 2008 ▶); software used to prepare material for publication: *SHELXTL*.

## Supplementary Material

Crystal structure: contains datablocks global, I. DOI: 10.1107/S1600536808032170/si2116sup1.cif

Structure factors: contains datablocks I. DOI: 10.1107/S1600536808032170/si2116Isup2.hkl

Additional supplementary materials:  crystallographic information; 3D view; checkCIF report

## Figures and Tables

**Table 1:** Hydrogen-bond geometry (Å, °)

*D*—H⋯*A*	*D*—H	H⋯*A*	*D*⋯*A*	*D*—H⋯*A*
O1—H1⋯O2^i^	0.82	1.82	2.632 (4)	174

Symmetry code: (i) 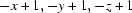.

**Fig. 1.**
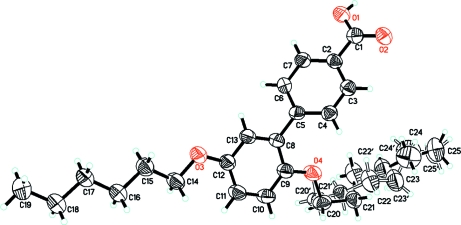
The molecular structure of the title compound with displacement ellipsoids at the 30% probability level. The disordered components are shown.

**Fig. 2.**
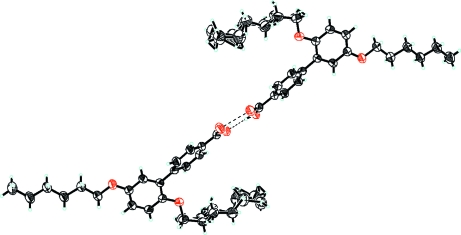
The dimeric structure of the title compound linked by the O—H···O hydrogen bonds.

## supplementary crystallographic information

### Comment

Palladium-catalyzed Suzuki coupling reaction has become an extremely powerful method in synthesis for the formation of carbon-carbon bond (Kotha *et al.*, 2002). For example, 1,4-Dibromide-2,5-bis(hexyloxy)benzene was reacted with 4-carboxyphenylboronic acid in the presence of Pd(PPh_3_)_4_ to give coupling product 2,5-bis(hexyloxy)-1,4-di(4'-carboxyphenyl)benzene (Zhang *et al.*, 2006). In the above reaction, we obtained the title compound as a side product.

A view of the molecular structure of the title compound is given in Fig.1. The dihedral angle between benzene rings is 44.5 (3)°. One n-hexyl chain of the hexyloxyl group has the same fully extended all - *trans* conformation as the 1,4-Dibromide-2,5-bis(hexyloxy)benzene (Li, *et al.*, 2008), while the other n-hexyl chain displays disorder with site occupancies 0.470 (3) and 0.530 (3). In the crystal structure, centrosymmetric dimers arise from pairs of O—H···O hydrogen bonds involving the carboxylic acid groups (Fig.2, Table 1).

### Experimental

1,4-Dibromo-2,5-bis(hexyloxy)benzene was prepared as described in the literature (Maruyama & Kawanishi 2002). The title compound was obtained as a side-product from the Suzuki coupling reaction of 1,4-Dibromo-2,5-bis(hexyloxy)benzene and 4-carboxyphenylboronic acid as described in the literature (Zhang *et al.*, 2006) and recrystallized from ethanol at room temperature to give the desired crystals suitable for single-crystal X-ray diffraction.

### Refinement

H atoms attached to C atoms of the title compound were placed in geometrically idealized positions and treated as riding with C—H distances constrained to 0.93 (aromatic CH), or 0.96 Å (methyl CH_3_), and 0.97 Å (methylene CH_2_) and constrained to ride on their parent atoms, with *U*_iso_(H) = 1.2 *U*_eq_(C) (1.5*U*_eq_ for methyl H).

### Crystal data

**Table:**

C_25_H_34_O_4_	*F*(000) = 864
*M**_r_* = 398.52	*D*_x_ = 1.121 Mg m^−^^3^
Monoclinic, *P*2_1_/*n*	Mo *K*α radiation, λ = 0.71073 Å
*a* = 7.2936 (12) Å	Cell parameters from 918 reflections
*b* = 14.689 (2) Å	θ = 2.8–17.8°
*c* = 22.137 (4) Å	µ = 0.07 mm^−^^1^
β = 95.283 (3)°	*T* = 295 K
*V* = 2361.7 (7) Å^3^	Block, colourless
*Z* = 4	0.35 × 0.15 × 0.06 mm

### Data collection

**Table:**

Bruker SMART CCD diffractometer	4359 independent reflections
Radiation source: fine-focus sealed tube	1432 reflections with *I* > 2σ(*I*)
graphite	*R*_int_ = 0.087
φ and ω scans	θ_max_ = 25.5°, θ_min_ = 2.3°
Absorption correction: multi-scan (SADABS; Sheldrick, 1996)	*h* = −8→8
*T*_min_ = 0.975, *T*_max_ = 0.996	*k* = −17→17
15777 measured reflections	*l* = −26→26

### Refinement

**Table:**

Refinement on *F*^2^	Secondary atom site location: difference Fourier map
Least-squares matrix: full	Hydrogen site location: inferred from neighbouring sites
*R*[*F*^2^ > 2σ(*F*^2^)] = 0.061	H-atom parameters constrained
*wR*(*F*^2^) = 0.194	*w* = 1/[σ^2^(*F*_o_^2^) + (0.0705*P*)^2^] where *P* = (*F*_o_^2^ + 2*F*_c_^2^)/3
*S* = 0.97	(Δ/σ)_max_ < 0.001
4359 reflections	Δρ_max_ = 0.16 e Å^−^^3^
257 parameters	Δρ_min_ = −0.14 e Å^−^^3^
0 restraints	Extinction correction: SHELXL97 (Sheldrick, 2008), Fc^*^=kFc[1+0.001xFc^2^λ^3^/sin(2θ)]^-1/4^
Primary atom site location: structure-invariant direct methods	Extinction coefficient: 0.0061 (13)

### Special details

**Table:**

Geometry. All e.s.d.'s (except the e.s.d. in the dihedral angle between two l.s. planes)are estimated using the full covariance matrix. The cell e.s.d.'s are takeninto account individually in the estimation of e.s.d.'s in distances, anglesand torsion angles; correlations between e.s.d.'s in cell parameters are onlyused when they are defined by crystal symmetry. An approximate (isotropic)treatment of cell e.s.d.'s is used for estimating e.s.d.'s involving l.s. planes.
Refinement. Refinement of *F*^2^ against ALL reflections. The weighted *R*-factor *wR* and goodness of fit *S* are based on *F*^2^, conventional *R*-factors *R* are based on *F*, with *F* set to zero for negative *F*^2^. The threshold expression of *F*^2^ > σ(*F*^2^) is used only for calculating *R*-factors(gt) *etc*. and is not relevant to the choice of reflections for refinement. *R*-factors based on *F*^2^ are statistically about twice as large as those based on *F*, and *R*- factors based on ALL data will be even larger.

### Fractional atomic coordinates and isotropic or equivalent isotropic displacement parameters (Å^2^)

**Table:**

	*x*	*y*	*z*	*U*_iso_*/*U*_eq_	Occ. (<1)
C20	0.7088 (7)	0.0256 (3)	0.2574 (2)	0.1007 (16)	0.470 (3)
H20A	0.7986	−0.0208	0.2703	0.121*	0.470 (3)
H20B	0.6837	0.0216	0.2136	0.121*	0.470 (3)
C21	0.5320 (5)	0.0098 (4)	0.28753 (19)	0.108 (3)	0.470 (3)
H21A	0.4350	0.0476	0.2679	0.129*	0.470 (3)
H21B	0.4949	−0.0533	0.2821	0.129*	0.470 (3)
C22	0.5551 (5)	0.0315 (5)	0.35456 (16)	0.137 (4)	0.470 (3)
H22A	0.5929	0.0946	0.3594	0.165*	0.470 (3)
H22B	0.6542	−0.0058	0.3734	0.165*	0.470 (3)
C23	0.3866 (6)	0.0171 (4)	0.3885 (2)	0.155 (4)	0.470 (3)
H23A	0.3371	−0.0429	0.3787	0.187*	0.470 (3)
H23B	0.4241	0.0183	0.4317	0.187*	0.470 (3)
C24	0.2371 (8)	0.0856 (3)	0.3751 (2)	0.162 (3)	0.470 (3)
H24A	0.1901	0.0795	0.3328	0.195*	0.470 (3)
H24B	0.2902	0.1460	0.3803	0.195*	0.470 (3)
C25	0.0785 (8)	0.0786 (4)	0.4134 (2)	0.153 (3)	0.470 (3)
H25A	−0.0192	0.0445	0.3920	0.229*	0.470 (3)
H25B	0.0353	0.1386	0.4219	0.229*	0.470 (3)
H25C	0.1180	0.0483	0.4508	0.229*	0.470 (3)
C20'	0.7495 (6)	0.0177 (3)	0.2742 (2)	0.1007 (16)	0.530 (3)
H20C	0.8659	−0.0135	0.2835	0.121*	0.530 (3)
H20D	0.6987	−0.0016	0.2342	0.121*	0.530 (3)
C21'	0.6199 (6)	−0.0085 (3)	0.3200 (2)	0.108 (3)	0.530 (3)
H21C	0.5838	−0.0714	0.3129	0.129*	0.530 (3)
H21D	0.6861	−0.0053	0.3600	0.129*	0.530 (3)
C22'	0.4489 (5)	0.0478 (2)	0.32045 (16)	0.137 (4)	0.530 (3)
H22C	0.4839	0.1083	0.3347	0.165*	0.530 (3)
H22D	0.3942	0.0537	0.2790	0.165*	0.530 (3)
C23'	0.3055 (5)	0.0122 (3)	0.3584 (2)	0.155 (4)	0.530 (3)
H23C	0.1984	−0.0048	0.3315	0.187*	0.530 (3)
H23D	0.3527	−0.0427	0.3785	0.187*	0.530 (3)
C24'	0.2443 (6)	0.0752 (3)	0.4053 (2)	0.162 (3)	0.530 (3)
H24C	0.2090	0.1325	0.3858	0.195*	0.530 (3)
H24D	0.3487	0.0873	0.4346	0.195*	0.530 (3)
C25'	0.0875 (7)	0.0431 (4)	0.4391 (3)	0.153 (3)	0.530 (3)
H25D	0.0275	−0.0073	0.4180	0.229*	0.530 (3)
H25E	0.0009	0.0918	0.4419	0.229*	0.530 (3)
H25F	0.1336	0.0242	0.4792	0.229*	0.530 (3)
O1	0.7303 (4)	0.4749 (2)	0.48474 (13)	0.1015 (9)	
H1	0.6643	0.4951	0.5097	0.152*	
O2	0.4582 (4)	0.45546 (17)	0.43055 (11)	0.0937 (9)	
O3	1.3854 (4)	0.25963 (18)	0.17662 (11)	0.0957 (9)	
O4	0.7809 (4)	0.11432 (17)	0.27390 (12)	0.1056 (10)	
C1	0.6290 (7)	0.4451 (3)	0.43801 (19)	0.0811 (12)	
C2	0.7271 (6)	0.3934 (2)	0.39327 (17)	0.0735 (10)	
C3	0.6246 (5)	0.3488 (3)	0.34666 (18)	0.0766 (11)	
H3	0.4969	0.3535	0.3432	0.092*	
C4	0.7099 (6)	0.2974 (2)	0.30537 (16)	0.0774 (11)	
H4	0.6384	0.2684	0.2741	0.093*	
C5	0.9002 (6)	0.2878 (2)	0.30924 (16)	0.0692 (10)	
C6	1.0009 (5)	0.3342 (3)	0.35605 (18)	0.0835 (12)	
H6	1.1287	0.3303	0.3593	0.100*	
C7	0.9163 (6)	0.3863 (3)	0.39816 (17)	0.0832 (11)	
H7	0.9869	0.4160	0.4293	0.100*	
C8	0.9934 (5)	0.2331 (3)	0.26465 (15)	0.0720 (10)	
C9	0.9291 (6)	0.1468 (3)	0.24652 (17)	0.0810 (11)	
C10	1.0170 (6)	0.0979 (3)	0.20426 (17)	0.0909 (12)	
H10	0.9734	0.0406	0.1923	0.109*	
C11	1.1685 (6)	0.1332 (3)	0.17971 (17)	0.0892 (12)	
H11	1.2252	0.1001	0.1509	0.107*	
C12	1.2361 (6)	0.2173 (3)	0.19762 (17)	0.0791 (11)	
C13	1.1469 (5)	0.2661 (3)	0.23992 (15)	0.0761 (11)	
H13	1.1922	0.3231	0.2520	0.091*	
C14	1.4854 (6)	0.2112 (3)	0.13414 (17)	0.0936 (12)	
H14A	1.4047	0.1959	0.0983	0.112*	
H14B	1.5351	0.1552	0.1522	0.112*	
C15	1.6382 (5)	0.2718 (3)	0.11761 (17)	0.0939 (12)	
H15A	1.7197	0.2842	0.1538	0.113*	
H15B	1.5863	0.3293	0.1029	0.113*	
C16	1.7496 (6)	0.2318 (3)	0.06986 (17)	0.1004 (13)	
H16A	1.6698	0.2225	0.0328	0.120*	
H16B	1.7964	0.1727	0.0835	0.120*	
C17	1.9103 (6)	0.2919 (3)	0.05617 (17)	0.0987 (13)	
H17A	1.8627	0.3502	0.0413	0.118*	
H17B	1.9872	0.3028	0.0936	0.118*	
C18	2.0261 (6)	0.2524 (3)	0.01070 (19)	0.1182 (16)	
H18A	1.9485	0.2401	−0.0264	0.142*	
H18B	2.0757	0.1947	0.0260	0.142*	
C19	2.1840 (6)	0.3126 (3)	−0.0042 (2)	0.1344 (18)	
H19A	2.1360	0.3676	−0.0230	0.202*	
H19B	2.2569	0.2812	−0.0315	0.202*	
H19C	2.2591	0.3273	0.0324	0.202*	

### Atomic displacement parameters (Å^2^)

**Table:**

	*U*^11^	*U*^22^	*U*^33^	*U*^12^	*U*^13^	*U*^23^
C20	0.121 (4)	0.072 (3)	0.111 (4)	−0.021 (3)	0.019 (3)	−0.011 (3)
C21	0.089 (7)	0.075 (4)	0.163 (8)	0.001 (4)	0.031 (6)	−0.007 (5)
C22	0.124 (8)	0.105 (5)	0.191 (10)	0.015 (5)	0.057 (7)	0.039 (6)
C23	0.119 (7)	0.115 (5)	0.241 (10)	0.004 (5)	0.063 (7)	0.002 (6)
C24	0.217 (8)	0.172 (7)	0.096 (7)	0.008 (6)	0.006 (6)	0.023 (6)
C25	0.112 (5)	0.173 (8)	0.175 (8)	0.034 (5)	0.020 (5)	0.026 (7)
C20'	0.121 (4)	0.072 (3)	0.111 (4)	−0.021 (3)	0.019 (3)	−0.011 (3)
C21'	0.089 (7)	0.075 (4)	0.163 (8)	0.001 (4)	0.031 (6)	−0.007 (5)
C22'	0.124 (8)	0.105 (5)	0.191 (10)	0.015 (5)	0.057 (7)	0.039 (6)
C23'	0.119 (7)	0.115 (5)	0.241 (10)	0.004 (5)	0.063 (7)	0.002 (6)
C24'	0.217 (8)	0.172 (7)	0.096 (7)	0.008 (6)	0.006 (6)	0.023 (6)
C25'	0.112 (5)	0.173 (8)	0.175 (8)	0.034 (5)	0.020 (5)	0.026 (7)
O1	0.102 (2)	0.112 (2)	0.094 (2)	−0.0008 (18)	0.0274 (17)	−0.0269 (18)
O2	0.095 (2)	0.103 (2)	0.0856 (19)	0.0112 (18)	0.0216 (17)	−0.0026 (16)
O3	0.099 (2)	0.097 (2)	0.0964 (19)	−0.0086 (17)	0.0363 (17)	−0.0176 (16)
O4	0.120 (2)	0.0675 (18)	0.137 (2)	−0.0163 (17)	0.052 (2)	−0.0183 (17)
C1	0.096 (4)	0.082 (3)	0.069 (3)	−0.008 (3)	0.022 (3)	0.002 (2)
C2	0.084 (3)	0.069 (2)	0.070 (3)	−0.001 (2)	0.023 (2)	−0.003 (2)
C3	0.080 (3)	0.074 (3)	0.077 (3)	0.000 (2)	0.017 (2)	0.003 (2)
C4	0.081 (3)	0.075 (3)	0.078 (3)	−0.007 (2)	0.011 (2)	−0.002 (2)
C5	0.080 (3)	0.057 (2)	0.071 (3)	−0.001 (2)	0.009 (2)	−0.001 (2)
C6	0.070 (3)	0.089 (3)	0.093 (3)	0.001 (2)	0.017 (2)	−0.012 (2)
C7	0.087 (3)	0.087 (3)	0.078 (3)	−0.006 (2)	0.017 (2)	−0.014 (2)
C8	0.083 (3)	0.063 (3)	0.071 (2)	0.004 (2)	0.014 (2)	−0.007 (2)
C9	0.094 (3)	0.068 (3)	0.084 (3)	−0.004 (2)	0.023 (2)	−0.005 (2)
C10	0.120 (4)	0.069 (3)	0.087 (3)	−0.010 (3)	0.024 (3)	−0.014 (2)
C11	0.108 (3)	0.085 (3)	0.078 (3)	−0.001 (3)	0.026 (2)	−0.013 (2)
C12	0.083 (3)	0.077 (3)	0.080 (3)	−0.002 (3)	0.017 (2)	−0.005 (2)
C13	0.084 (3)	0.071 (3)	0.075 (2)	0.002 (2)	0.012 (2)	−0.012 (2)
C14	0.106 (3)	0.093 (3)	0.086 (3)	0.005 (3)	0.031 (3)	−0.012 (2)
C15	0.095 (3)	0.100 (3)	0.089 (3)	0.004 (3)	0.021 (3)	−0.004 (3)
C16	0.099 (3)	0.110 (3)	0.096 (3)	0.002 (3)	0.028 (3)	−0.020 (3)
C17	0.100 (3)	0.104 (3)	0.096 (3)	0.002 (3)	0.031 (3)	−0.016 (2)
C18	0.110 (4)	0.129 (4)	0.121 (4)	0.001 (3)	0.041 (3)	−0.030 (3)
C19	0.117 (4)	0.154 (5)	0.139 (4)	−0.019 (3)	0.049 (3)	−0.025 (3)

### Geometric parameters (Å, °)

**Table:**

C20—O4	1.440 (5)	O3—C14	1.432 (4)
C20—C21	1.5236 (17)	O4—C9	1.372 (4)
C20—H20A	0.9700	C1—C2	1.483 (5)
C20—H20B	0.9700	C2—C7	1.378 (4)
C21—C22	1.5119 (17)	C2—C3	1.383 (4)
C21—H21A	0.9700	C3—C4	1.378 (4)
C21—H21B	0.9700	C3—H3	0.9300
C22—C23	1.5129 (17)	C4—C5	1.390 (4)
C22—H22A	0.9700	C4—H4	0.9300
C22—H22B	0.9700	C5—C6	1.392 (4)
C23—C24	1.4930 (17)	C5—C8	1.486 (4)
C23—H23A	0.9700	C6—C7	1.393 (4)
C23—H23B	0.9700	C6—H6	0.9300
C24—C25	1.4994 (17)	C7—H7	0.9300
C24—H24A	0.9700	C8—C13	1.379 (4)
C24—H24B	0.9700	C8—C9	1.397 (5)
C25—H25A	0.9600	C9—C10	1.383 (5)
C25—H25B	0.9600	C10—C11	1.377 (5)
C25—H25C	0.9600	C10—H10	0.9300
C20'—O4	1.438 (4)	C11—C12	1.374 (5)
C20'—C21'	1.4978 (17)	C11—H11	0.9300
C20'—H20C	0.9700	C12—C13	1.388 (4)
C20'—H20D	0.9700	C13—H13	0.9300
C21'—C22'	1.4966 (17)	C14—C15	1.498 (5)
C21'—H21C	0.9700	C14—H14A	0.9700
C21'—H21D	0.9700	C14—H14B	0.9700
C22'—C23'	1.4938 (17)	C15—C16	1.510 (4)
C22'—H22C	0.9700	C15—H15A	0.9700
C22'—H22D	0.9700	C15—H15B	0.9700
C23'—C24'	1.4906 (17)	C16—C17	1.520 (5)
C23'—H23C	0.9700	C16—H16A	0.9700
C23'—H23D	0.9700	C16—H16B	0.9700
C24'—C25'	1.4994 (17)	C17—C18	1.490 (4)
C24'—H24C	0.9700	C17—H17A	0.9700
C24'—H24D	0.9700	C17—H17B	0.9700
C25'—H25D	0.9600	C18—C19	1.513 (5)
C25'—H25E	0.9600	C18—H18A	0.9700
C25'—H25F	0.9600	C18—H18B	0.9700
O1—C1	1.291 (4)	C19—H19A	0.9600
O1—H1	0.8200	C19—H19B	0.9600
O2—C1	1.251 (4)	C19—H19C	0.9600
O3—C12	1.372 (4)		
			
O4—C20—C21	109.3 (2)	C7—C2—C3	119.4 (4)
O4—C20—H20A	109.8	C7—C2—C1	121.8 (4)
C21—C20—H20A	109.8	C3—C2—C1	118.7 (4)
O4—C20—H20B	109.8	C4—C3—C2	120.6 (4)
C21—C20—H20B	109.8	C4—C3—H3	119.7
H20A—C20—H20B	108.3	C2—C3—H3	119.7
C22—C21—C20	112.1	C3—C4—C5	121.6 (4)
C22—C21—H21A	109.2	C3—C4—H4	119.2
C20—C21—H21A	109.2	C5—C4—H4	119.2
C22—C21—H21B	109.2	C4—C5—C6	116.9 (3)
C20—C21—H21B	109.2	C4—C5—C8	121.9 (4)
H21A—C21—H21B	107.9	C6—C5—C8	121.2 (4)
C21—C22—C23	115.8	C5—C6—C7	122.1 (4)
C21—C22—H22A	108.3	C5—C6—H6	119.0
C23—C22—H22A	108.3	C7—C6—H6	119.0
C21—C22—H22B	108.3	C2—C7—C6	119.4 (4)
C23—C22—H22B	108.3	C2—C7—H7	120.3
H22A—C22—H22B	107.4	C6—C7—H7	120.3
C24—C23—C22	114.8	C13—C8—C9	118.0 (3)
C24—C23—H23A	108.6	C13—C8—C5	120.7 (4)
C22—C23—H23A	108.6	C9—C8—C5	121.3 (4)
C24—C23—H23B	108.6	O4—C9—C10	123.4 (4)
C22—C23—H23B	108.6	O4—C9—C8	116.5 (4)
H23A—C23—H23B	107.5	C10—C9—C8	120.1 (4)
C23—C24—C25	115.4	C11—C10—C9	120.7 (4)
C23—C24—H24A	108.4	C11—C10—H10	119.7
C25—C24—H24A	108.4	C9—C10—H10	119.7
C23—C24—H24B	108.4	C12—C11—C10	120.3 (4)
C25—C24—H24B	108.4	C12—C11—H11	119.9
H24A—C24—H24B	107.5	C10—C11—H11	119.9
O4—C20'—C21'	111.6 (2)	O3—C12—C11	125.8 (4)
O4—C20'—H20C	109.3	O3—C12—C13	115.5 (4)
C21'—C20'—H20C	109.3	C11—C12—C13	118.7 (4)
O4—C20'—H20D	109.3	C8—C13—C12	122.3 (4)
C21'—C20'—H20D	109.3	C8—C13—H13	118.9
H20C—C20'—H20D	108.0	C12—C13—H13	118.9
C22'—C21'—C20'	116.2	O3—C14—C15	107.5 (3)
C22'—C21'—H21C	108.2	O3—C14—H14A	110.2
C20'—C21'—H21C	108.2	C15—C14—H14A	110.2
C22'—C21'—H21D	108.2	O3—C14—H14B	110.2
C20'—C21'—H21D	108.2	C15—C14—H14B	110.2
H21C—C21'—H21D	107.4	H14A—C14—H14B	108.5
C23'—C22'—C21'	116.0	C14—C15—C16	113.6 (4)
C23'—C22'—H22C	108.3	C14—C15—H15A	108.9
C21'—C22'—H22C	108.3	C16—C15—H15A	108.9
C23'—C22'—H22D	108.3	C14—C15—H15B	108.9
C21'—C22'—H22D	108.3	C16—C15—H15B	108.9
H22C—C22'—H22D	107.4	H15A—C15—H15B	107.7
C24'—C23'—C22'	116.4	C15—C16—C17	112.8 (3)
C24'—C23'—H23C	108.2	C15—C16—H16A	109.0
C22'—C23'—H23C	108.2	C17—C16—H16A	109.0
C24'—C23'—H23D	108.2	C15—C16—H16B	109.0
C22'—C23'—H23D	108.2	C17—C16—H16B	109.0
H23C—C23'—H23D	107.3	H16A—C16—H16B	107.8
C23'—C24'—C25'	116.4	C18—C17—C16	113.8 (4)
C23'—C24'—H24C	108.2	C18—C17—H17A	108.8
C25'—C24'—H24C	108.2	C16—C17—H17A	108.8
C23'—C24'—H24D	108.2	C18—C17—H17B	108.8
C25'—C24'—H24D	108.2	C16—C17—H17B	108.8
H24C—C24'—H24D	107.4	H17A—C17—H17B	107.7
C24'—C25'—H25D	109.5	C17—C18—C19	114.2 (4)
C24'—C25'—H25E	109.5	C17—C18—H18A	108.7
H25D—C25'—H25E	109.5	C19—C18—H18A	108.7
C24'—C25'—H25F	109.5	C17—C18—H18B	108.7
H25D—C25'—H25F	109.5	C19—C18—H18B	108.7
H25E—C25'—H25F	109.5	H18A—C18—H18B	107.6
C1—O1—H1	109.5	C18—C19—H19A	109.5
C12—O3—C14	117.7 (3)	C18—C19—H19B	109.5
C9—O4—C20'	118.6 (3)	H19A—C19—H19B	109.5
C9—O4—C20	119.1 (3)	C18—C19—H19C	109.5
O2—C1—O1	123.6 (4)	H19A—C19—H19C	109.5
O2—C1—C2	120.7 (4)	H19B—C19—H19C	109.5
O1—C1—C2	115.7 (4)		
			
O4—C20—C21—C22	−49.3 (6)	C6—C5—C8—C13	−43.6 (5)
C20—C21—C22—C23	−179.6	C4—C5—C8—C9	−45.5 (5)
C21—C22—C23—C24	−72.3	C6—C5—C8—C9	136.4 (4)
C22—C23—C24—C25	−173.4	C20'—O4—C9—C10	20.1 (6)
O4—C20'—C21'—C22'	46.6 (6)	C20—O4—C9—C10	−1.4 (6)
C20'—C21'—C22'—C23'	169.9	C20'—O4—C9—C8	−158.2 (3)
C21'—C22'—C23'—C24'	124.5	C20—O4—C9—C8	−179.7 (3)
C22'—C23'—C24'—C25'	174.2	C13—C8—C9—O4	177.3 (3)
C21'—C20'—O4—C9	163.6 (3)	C5—C8—C9—O4	−2.6 (5)
C21'—C20'—O4—C20	−99.5 (2)	C13—C8—C9—C10	−1.0 (5)
C21—C20—O4—C9	−173.0 (3)	C5—C8—C9—C10	179.1 (3)
C21—C20—O4—C20'	93.5 (2)	O4—C9—C10—C11	−178.1 (3)
O2—C1—C2—C7	175.5 (4)	C8—C9—C10—C11	0.1 (6)
O1—C1—C2—C7	−6.7 (5)	C9—C10—C11—C12	1.0 (6)
O2—C1—C2—C3	−6.8 (5)	C14—O3—C12—C11	−2.8 (5)
O1—C1—C2—C3	171.1 (3)	C14—O3—C12—C13	178.3 (3)
C7—C2—C3—C4	0.1 (5)	C10—C11—C12—O3	180.0 (3)
C1—C2—C3—C4	−177.7 (3)	C10—C11—C12—C13	−1.1 (6)
C2—C3—C4—C5	0.6 (5)	C9—C8—C13—C12	0.9 (5)
C3—C4—C5—C6	−1.3 (5)	C5—C8—C13—C12	−179.2 (3)
C3—C4—C5—C8	−179.6 (3)	O3—C12—C13—C8	179.2 (3)
C4—C5—C6—C7	1.4 (5)	C11—C12—C13—C8	0.2 (5)
C8—C5—C6—C7	179.7 (3)	C12—O3—C14—C15	179.1 (3)
C3—C2—C7—C6	0.0 (5)	O3—C14—C15—C16	−176.5 (3)
C1—C2—C7—C6	177.7 (3)	C14—C15—C16—C17	−176.9 (3)
C5—C6—C7—C2	−0.8 (5)	C15—C16—C17—C18	178.0 (3)
C4—C5—C8—C13	134.6 (4)	C16—C17—C18—C19	178.7 (4)

### Hydrogen-bond geometry (Å, °)

**Table:**

*D*—H···*A*	*D*—H	H···*A*	*D*···*A*	*D*—H···*A*
O1—H1···O2^i^	0.82	1.82	2.632 (4)	174

Symmetry codes: (i) −*x*+1, −*y*+1, −*z*+1.
